# Arbitration between model-free and model-based control is not affected by transient changes in tonic serotonin levels

**DOI:** 10.1177/02698811231216325

**Published:** 2023-12-27

**Authors:** Maximilian D. Gilger, Lydia Hellrung, Philipp T. Neukam, Nils B. Kroemer, Stephan Nebe, Shakoor Pooseh, Yacila I. Deza-Lougovski, Michael N. Smolka

**Affiliations:** 1 Department of Psychiatry and Psychotherapy, Technische Universität Dresden, Dresden, Germany; 2 Zurich Center for Neuroeconomics, Department of Economics, University of Zurich, Zurich, Switzerland; 3 Depression and Anxiety Center for Discovery and Treatment, Department of Psychiatry, Icahn School of Medicine at Mount Sinai, New York, NY, USA; 4 Section of Medical Psychology, Department of Psychiatry and Psychotherapy, Faculty of Medicine, University of Bonn, Bonn, Germany; 5 Department of Psychiatry and Psychotherapy, Tübingen Center for Mental Health, University of Tübingen, Tübingen, Germany; 6 German Center for Mental Health (DZPG), Tübingen, Germany; 7 Freiburg Center for Data Analysis and Modelling, Institute of Physics, University of Freiburg, Freiburg, Germany; 8 Institute of Psychology, University of the Bundeswehr München, Neubiberg, Germany

**Keywords:** Tryptophan loading, tryptophan depletion, habitual system, goal-directed system, two-stage Markov decision task

## Abstract

**Background:**

Serotonin has been suggested to modulate decision-making by influencing the arbitration between model-based and model-free control. Disruptions in these control mechanisms are involved in mental disorders such as drug dependence or obsessive-compulsive disorder. While previous reports indicate that lower brain serotonin levels reduce model-based control, it remains unknown whether increases in serotonergic availability might thus increase model-based control. Moreover, the mediating neural mechanisms have not been studied yet.

**Aim:**

The first aim of this study was to investigate whether increased/decreased tonic serotonin levels affect the arbitration between model-free and model-based control. Second, we aimed to identify the underlying neural processes.

**Methods:**

We employed a sequential two-stage Markov decision-task and measured brain responses during functional magnetic resonance imaging in 98 participants in a randomized, double-blind cross-over within-subject design. To investigate the influence of serotonin on the balance between model-free and model-based control, we used a tryptophan intervention with three intervention levels (loading, balanced, depletion). We hypothesized that model-based behaviour would increase with higher serotonin levels.

**Results:**

We found evidence that neither model-free nor model-based control were affected by changes in tonic serotonin levels. Furthermore, our tryptophan intervention did not elicit relevant changes in Blood-Oxygenation-Level Dependent activity.

## Introduction

During our everyday life, we have to make decisions regularly. Sometimes these decisions involve careful consideration of the awaited outcomes and a constant revaluation of our behaviour. Some other decisions are more driven by our daily routine and are thereby more efficient in terms of time and cognitive load. Every decision entails costs and benefits that are typically on different timescales and impose different amounts of cognitive load. The aim of our decisions is always to find the most promising option to maximize pleasant outcomes and to avoid negative consequences. At least two distinct control mechanisms are involved in the process of decision making: the goal-directed (model-based) and the habitual (model-free) system (Daw et al., 2005; Dickinson and Balleine, 2002; Dolan and Dayan, 2013). Both systems are moderated by multiple neurotransmitters, that is, serotonin and dopamine. Disruptions in either serotonergic and dopaminergic systems have been found to be involved in various mental disorders where impairments in decision making processes are a landmark such as drug dependence (Corbit et al., 2012; Garbusow et al., 2014; Leong et al., 2016), schizophrenia (cf. Griffiths et al., 2014; Morris et al., 2015) or obsessive compulsive disorder (Gillan et al., 2011, 2016; Voon et al., 2014).

The habitual system relies on Thorndike’s ‘Law of Effect’ (Thorndike, 1911), which states that a rewarded action is more likely to be repeated in the future. With repetition, the habitual system builds stimulus–action associations (Balleine et al., 2009). Especially after extensive training, these stimulus–action associations can become habitual. As a result, actions are no longer dependent on the contingency of their outcomes or their respective values (cf. Balleine et al., 2009; Balleine and O’Doherty, 2010), and might be repeated even without motivational relevance (Daw et al., 2005; Dolan and Dayan, 2013), which is highly relevant to explain addictive behaviours. The value assigned to such a stimulus–response pair is associated to the average value of previously obtained rewards. The difference between the expected and the actually received reward, the so-called reward prediction error (RPE) (Schultz et al., 1997), guides the value-learning of a stimulus–response pair. Consequently, the habitual system only requires the RPE and does not take any information about the current state of the environment into account – it is considered model-free (MF).

In contrast, the more flexible and prospective goal-directed system is based on an internal model of the environment, akin to a cognitive map (Tolman, 1948) and has, hence, been termed the model-based (MB) system (Balleine et al., 2009; Worbe et al., 2016). Goal-directed behaviour is characterized by (1) taking the relationship between an action and its outcome into account, and (2), in contrast to habitual behaviour, the outcome being motivationally relevant for the subject (Dolan and Dayan, 2013). Such a decision process is comparable to a search in the cognitive map, which aims to identify the most rewarding option at a given state of the environment. Although this mechanism is theoretically advantageous, the MB system requires a lot of resources and time for its computations of action–outcome associations, which poses a limitation in complex environments. For optimal behaviour, it is pivotal that these two systems are well balanced, but it remains unclear how this balance is achieved in the brain. One approach to assess the balance of MF and MB control is the so called two-stage task, a variant of the sequential Markov decision task (Daw et al., 2011).

An open question is whether and how serotonin modulates MB and MF control. One study used the slips-of-action task (De Wit et al., 2007) to assess goal-directed and habitual behaviour. They found that participants undergoing tryptophan depletion behaved more habitual compared to a control group (Worbe et al., 2015). In a second study the group used the original two-stage task (Daw et al., 2011) in which participants were to maximize monetary gains (reward version), and also a version where they were told to minimize monetary losses (punishment version). Using a between-group design, Worbe et al. found that tryptophan depletion impaired MB control and shifts behaviour to more habitual response patterns in the reward version (Worbe et al., 2016). On the contrary, no effect of the intervention was found when participants performed the punishment version of the two-stage task.

Both studies by Worbe et al. (2016, 2015) are in line with the computational framework by Cools et al. (2011), which suggests that the average reward rate is associated with tonic dopamine levels whereas the average punishment rate is associated with tonic serotonin levels. The difference between tonic dopamine and serotonin is thought to code the overall average outcome rate, indicating the net density of rewards in the environment and thus, the cost of waiting for rewards. The average outcome rate might neurochemically be reflected by tonic dopamine and serotonin levels in the brain. Consequently, lower serotonin (or higher dopamine) levels are associated with a higher average reward rate and vice versa. Keramati et al. (2011) suggested that the balance between habitual and goal-directed control can be conceptualized as a speed/accuracy trade-off between the faster but often less accurate MF system and the computationally demanding, slower but more accurate MB system. Thus, lower serotonin levels should lead to a shift to MF control, as shown before (Worbe et al., 2015,^2016^).

Although both studies by Worbe et al. showed comparable effects on the balance between habitual and goal-directed control, the neural underpinnings of serotonergic effects on both control systems have not been investigated so far. Serotonin might modulate the function of key nodes for MB and MF learning such as the ventral striatum, the ventromedial prefrontal cortex (vmPFC), and the dorsolateral prefrontal cortex (dlPFC). As it has been shown previously, the ventral striatum integrates MF and MB learning signals (Daw et al., 2011) while the vmPFC additionally encodes action–outcome associations (i.e. MB learning) (Balleine and O’Doherty, 2010; Daw et al., 2006; Valentin et al., 2007). The dlPFC is involved in abstract task and rule representation as well as coding of expected rewards and behavioural switching (Nee et al., 2013; Smittenaar et al., 2013; Wager et al., 2004; Wallis et al., 2001).

An elegant way to manipulate central serotonin levels is to reduce or increase the dietary supply of tryptophan, the only precursor for serotonin in the human body (Biskup et al., 2012; Dingerkus et al., 2012; Wurtman et al., 1980). The effectiveness of this intervention has been shown in a positron emission tomography study, tracing the metabolism of tryptophan (Nishizawa et al., 1997). Notably, there is evidence that a polymorphism in the serotonin transporter gene-linked polymorphic region (*5-HTTLPR*) can alter synaptic serotonin levels (Canli and Lesch, 2007; DenOuden et al., 2013; Roiser et al., 2006) and thereby might interfere with the effectiveness of our tryptophan intervention.

To extend previous findings, we investigated the behavioural and neural effects of serotonergic modulation on MF and MB behaviour during performance of the two-stage task in a functional magnetic resonance imaging (fMRI) study using an acute tryptophan depletion (ATD) and, for the first time, also an acute tryptophan loading (ATL) condition. To increase statistical power, we used a within-subject design comprising three sessions per participant. We hypothesized that higher serotonin levels after ATL would increase goal-directed MB behaviour, whereas lower serotonin levels after ATD should increase habitual MF behaviour. We reasoned that these behavioural changes should be reflected by an increased Blood-Oxygenation-Level Dependent (BOLD) activity in the vmPFC and dlPFC in the tryptophan loading condition. Contrariwise, the shift to more MF control in ATD should be reflected by an increased BOLD activity in the ventral striatum.

## Methods

### Participants

This study was part of the Collaborative Research Center 940 on cognition and volitional control, where these data have been acquired in the same sample as in a study investigating the serotonergic modulation of decision-making under risk (Neukam et al. (2018)), and inter-temporal choice behaviour (Neukam et al. 2019), as well as resting-state brain activity (Deza-Araujo et al. 2019). More details on participant recruitment are provided in the Supplemental Material. In short, we invited all eligible participants to an on-site baseline assessment for genotyping and a behavioural session. In total, 170 genotyped participants (Supplemental Figure S3(C) + (D)) took part in the main study. Due to organizational reasons and adverse reactions to the mixture, 107 participants finished all three sessions (Supplemental Figure S2). For analysis, we had to exclude five participants due to task unresponsiveness (more than 30% missing trials in at least one session), two due to technical issues during data acquisition, and two due to an intervention randomization error in one session. This resulted in a sample of *n* = 98 participants (37 female), aged 20 to 42 years (mean: 32.2 years, SD = 6.1 years) for behavioural analysis. For the fMRI analysis, we excluded another 10 participants due to insufficient data quality (see Supplemental Material) and hence analysed a sample of 88 participants (36 female). The excluded and remaining participants were equal regarding age, gender and education. All participants provided written informed consent prior to taking part in the study and received monetary compensation at the end of the study.

### Procedure and intervention

Briefly, all participants underwent four appointments: a baseline session for genotyping and behavioural testing and three fMRI sessions with three different tryptophan interventions to modulate neuronal serotonin levels using a randomized, double-blind cross-over study. For the amino acid mixture, we used the ‘Moja-De’ protocol (Dingerkus et al., 2012; Stewart et al., 2020; Zepf et al., 2008) in order to reduce negative side effects and prevent participants’ withdrawal from the study in comparison with other amino acid formulations (Young et al., 1985). All tryptophan interventions (amino acids mixtures) contained the same amount of large neutral amino acids (LNAAs) but differed in the amount of tryptophan. The amount of received amino acids was body-weight adapted (Biskup et al., 2012), with a total of 0.65 g/kg body weight of LNAAS. The depletion mixture (ATD) for low serotonin levels contained no tryptophan; for the balanced mixture (BAL) we added 0.007 g tryptophan/kg body weight and for the high-load mixture (ATL) we added 0.07 g tryptophan/kg body weight. Details on the amino acid mixture can be found in the Supplemental Table S2. To validate the effectiveness of the intervention, we sampled blood four times each session (see also Deza-Araujo et al., 2019; Neukam et al., 2018, 2019 and Supplemental Material). The study was approved by the institutional review boards of the Technische Universität Dresden in accordance with the Human Subjects Guidelines of the Declaration of Helsinki.

### Genotyping and analyses of blood tryptophan levels

The *5-HTTLPR* has a short (S) and a long (L) allele. We additionally genotyped the participants for a single nucleotide polymorphism (rs25531, adenine to guanine) in the L-allele of the *5-HTTLPR*, which resulted in a triallelic locus (S/L_A_/L_G_). The details of the genotyping procedure are described elsewhere (Dukal et al., 2015; Kobiella et al., 2011) and in Supplemental Figure S1. We determined the amount of tryptophan and the LNAAs in the blood samples and calculated a tryptophan/∑(LNAA) ratio as an indicator of central serotonin availability. As threonine, lysine and methionine are part of the formulation for other reasons, such as reduced side effects of the mixture, but are no tryptophan competitors, we excluded these three amino acids from the calculation of the LNAA sum. To estimate the effect of the intervention we calculated the area under the curve (AUC) of the tryptophan/∑(LNAA) ratio on four timepoints (T0, shortly before drinking the respective mixture; T1, on hour after drinking the mixture; T2, 3 h after drinking the mixture; T3, 6.5 h after drinking the mixture). The values were normalized to T0. Normalized AUC values were then entered into a repeated-measures analysis of variance (ANOVA) with intervention as a within-subject factor. Briefly, we found that tryptophan levels reached their peak value 3 h after loading or their minimum 3 h after depletion (see Supplemental Figure S3). For detailed results and the statistical values of the ANOVAs see the Supplemental Material. Essentially, all interventions differed significantly from each other in the expected directions so we can clearly assume that our intervention worked.

### Behavioural task

**Figure 1.**
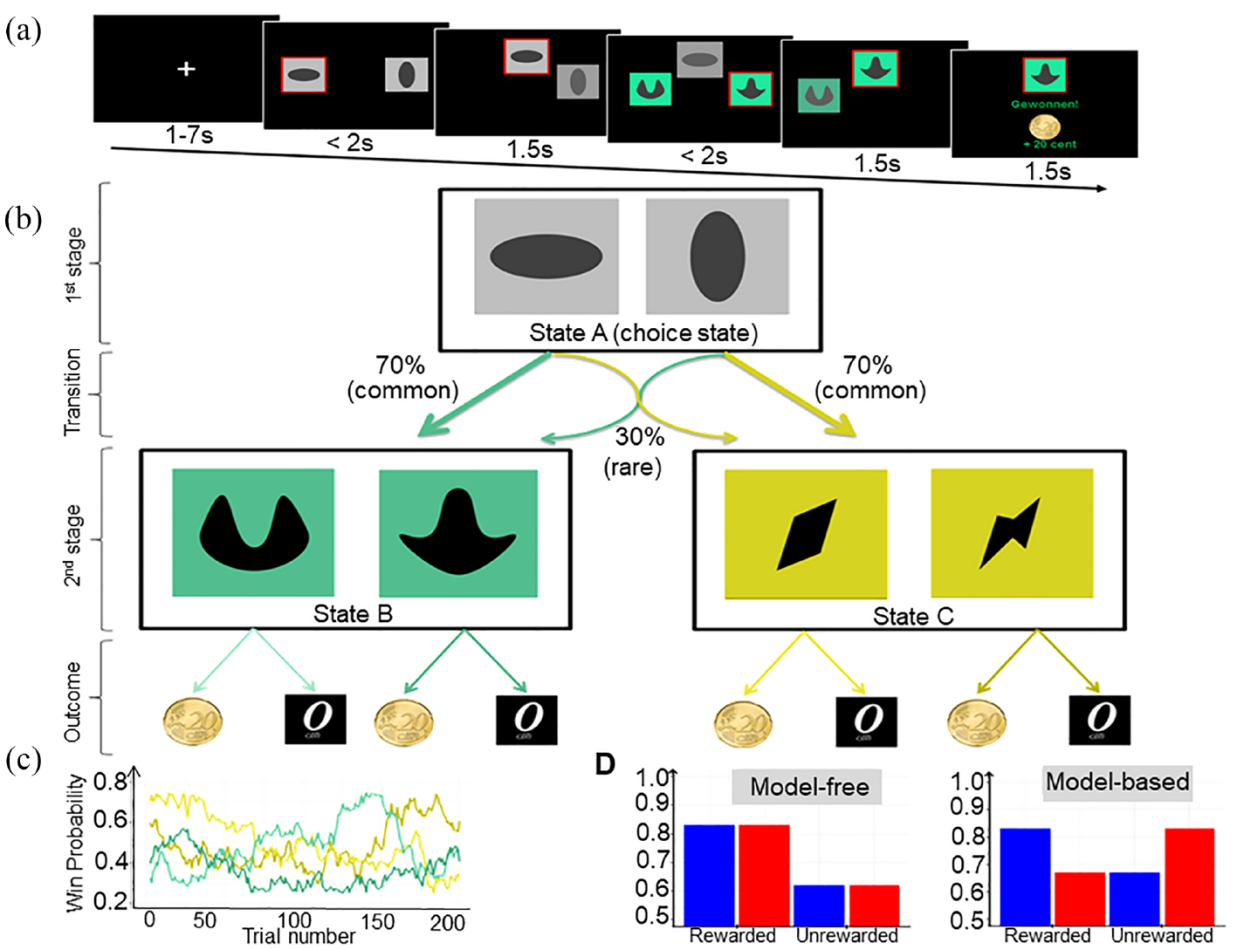
(a) Sequence of one trial: The chosen option is highlighted by a red frame and moved to the top of the screen. Then, the second step stimuli appear and can be chosen in the same manner. (b) Transition contingencies: Initially, participants had to choose between one of two offered grey boxes. In this case, the horizontal oval lead with a fixed transition probability of 70% to the turquoise pair of coloured boxes (common transition) and with a probability of 30% to the yellow pair of boxes (rare transition) and vice versa for the vertical oval. On second stage, participants had to select one of the coloured boxes to receive a reward or not (see c). Model-based (MB) participants consider the transition structure after receiving an outcome and before making the next decision. (c) Reward probability for second-stage options over time: The probability of a monetary reward after the second stage changes over time following a Gaussian random walk. (d) Expected response patterns for stay-switch probabilities: Under model-free control, the probabilities to repeat a first stage action are supposed to be higher for previously rewarded trials. In contrast, MB control integrates the transition structure, such that rewarded rare transitions do induce switching behaviour while unrewarded rare transitions do not lead to switching behaviour.

We used a two-stage sequential choice task as introduced by Daw et al. (2011), which was identical to the one previously used in our group (Chen et al., 2021; Nebe et al., 2018) (Figure 1). Each trial consisted of two sequential stages. At the first stage, the participants had to choose between two grey boxes that led them to one pair of coloured boxes (second-stage options, Figure 1(b)) with fixed transition probabilities (70% and 30%, respectively). At the second stage, participants had to decide for one of the two coloured boxes. The second choice was rewarded (20 cents) stochastically. The reward probabilities for the second stage options varied slowly according to Gaussian random walks with reflecting boundaries of 0.25 and 0.75, which encouraged learning throughout the task (Figure 1(c)). The task consisted of 201 trials in each session and took approximately 35 min. The goal for the participants was to identify and select the box with the highest reward probability at that time to maximize their monetary outcome. The most crucial features for the arbitration between MB and MF controls were (1) how strong first stage choices were driven by rewards obtained in the previous trial and (2) how strong this behaviour was affected by the transition structure between the sequential choices.

### Brain imaging

fMRI data acquisition started about 3.5 h after ingestion of the amino acids. The fMRI data was collected with a 3 Tesla Siemens Magnetom Trio Tim scanner (Siemens, Erlangen, Germany) equipped with a 32-channel head coil. During the in-scanner task, stimuli were presented using an fMRI compatible screen and rear-view mirror system. Participants responded by pressing their index fingers on two separate button boxes, one for each hand. Psychophysics Toolbox Version 3 (Brainard, 1997; Kleiner et al., 2007; Pelli, 1997) implemented in MATLAB (R2010a, The MathWorks Inc., Natick, MA﻿s, USA) was used for stimulus presentation. Functional images were acquired by using a gradient echo-planar imaging (EPI) sequence (repetition time (TR): 2410 ms; echo time (TE): 25 ms; flip angle: 80°; field of view: 192 × 192 mm^2^; matrix size: 64 × 64; voxel size: 3.0 × 3.0 × 2.0 mm^3^), with a 1 mm gap. Each volume consisted of 42 transverse slices acquired descending from the top, axially tilted parallel to the anterior commissure-posterior commissural line (total ~900 volumes for each participant, total scan time: ~35 min). A structural image was acquired using a T1-weighted magnetization prepared rapid acquisition with gradient echo sequence for anatomical localization as well as screening for structural abnormalities by a neuro-radiologist (TR: 1900 ms; TE: 2.52 ms; flip angle: 9°; field of view: 256 × 256 mm^2^; number of slices: 176; voxel size: 1.0 × 1.0 × 1.0 mm^3^). A fieldmap was recorded for distortion correction of the EPI images. All participants were given earplugs and small cushions to minimize head movements.

### Analysis of behavioural data

All statistical analyses were performed using SPSS 27 (IBM-SPSS, Chicago, IL, USA), JASP 0.14.1 (JASP Team; University of Amsterdam, Amsterdam, Netherlands) and Matlab R2012a (The MathWorks Inc., Natick, MA, USA) with a significance level of α = 0.05 (two-tailed).

#### Influence of tryptophan intervention on first stage choices

First, we examined the influence of the tryptophan intervention on the proportion to repeat the first-stage choice of the previous trial (stay probability) given the outcome (rewarded vs. unrewarded) and transition type (common vs. rare) of the previous trial. The main effect of reward and its interaction with transition reflect MF and MB behaviour, respectively (Daw et al., 2011). The MF effect was calculated by subtracting the mean of stay probabilities of trials that were previously unrewarded (after common and rare transitions) from the mean of stay probabilities after rewarded trials:

(1) $$\left(p_{\mathrm{com}+}+p_{\mathrm{rar}+}-p_{\mathrm{com}-}-p_{\mathrm{rar}-}\right)/2$$

The MB effect was calculated as:

(1) $$\left(p_{\mathrm{com}+}-p_{\mathrm{rar}+}-p_{\mathrm{com}-}+p_{\mathrm{rar}-}\right)/2$$

To analyse the effect of the tryptophan intervention, we set up a repeated-measures ANOVAs with the MF effect, and another with the MB effect as dependent variables and intervention as the factor of interest. Since *5-HTTLPR* genotype (DenOuden et al., 2013; He et al., 2010; Roiser et al., 2006) affects serotonergic functioning and effectiveness of a dietary tryptophan intervention (McBride et al., 1990; Nishizawa et al., 1997; Sambeth et al., 2007), we also investigated whether genotype is associated with behaviour in the two-stage task and whether it modulates the effect of the intervention. Therefore, we included the genotype information as a between-subject factor in our analysis. Furthermore, we added the order of the three interventions as between-subject factor to our ANOVA to adjust for possible carryover effects. As neither genotype nor order of the intervention were associated with the MF or MB effect (all *p* > 0.05), we decided in favour of clarity to report the results for the MB and MF effects alone.

#### Computational model

To describe the task behaviour in more detail, we applied the computational model introduced by Daw et al. (2011) to our data. The model consists of seven parameters in total, including an agent for MF and one for MB learning. The MF component learns via a state-action-reward-state-action temporal difference algorithm, which does not consider transition information for value updating. The MB component intends to maximize the expected value by taking the transition structure of the task into account. The final decision is made by a weighted combination of the MF and MB systems (hybrid reinforcement learning model). How strong each agent influences behaviour is determined by the weighting parameter omega. Additionally, the model computes a stage-skipping value update parameter lambda describing an update of first-stage values by the RPEs of the second stage, the parameter pi for the general first level repetition bias, and a learning rate alpha for first and second stage learning, respectively (see Supplemental Material for further details). The effect of the tryptophan intervention was tested via repeated-measures ANOVA for each of the seven model parameters, with intervention condition as a within-subject factor. We report the within-subject contrasts and the partial eta squared (denoted as η_p_^2^) as a measurement of effect size.

### Analysis of fMRI data

#### Preprocessing

Functional brain data were preprocessed using SPM8 (Wellcome Trust Centre for Neuroimaging, London, UK) implemented in Nipype Version 0.9.2 (Gorgolewski et al., 2011) and despiked with the ArtRepair toolbox (version 4) (Mazaika et al., 2009). Individual anatomical T1 images were co-registered to the individual mean EPI images before segmentation and normalization to Montreal Neurological Institute (MNI) space. The resulting transformation parameters were applied to the distortion-corrected, despiked, scan-to-scan motion (threshold: *T* = 0.5 mm/TR) and slice-time corrected EPI images to spatially normalize them to MNI space (resampled to the final voxel size: 2 × 2 × 2 mm^3^). Normalized EPI images were spatially smoothed with an isotropic Gaussian kernel (full width at half maximum: 8 mm). First- and second-level analyses were done with SPM8. During first-level analyses, data was high-pass-filtered at 128 s. We used an explicit whole-brain mask for the first-level analyses.

#### First-level statistics

Our first-level statistics general linear model was based on the factorial design reflecting the stay-switch behaviour of the participants (Kroemer et al., 2019). Neural processes at first stage onset were of special interest for us, because at this stage participants may have adapted their behaviour considering the previous reward and transition. We used previous reward (unrewarded/rewarded coded as −0.5/0.5), previous transition (rare/common as −0.5/0.5) and their interaction as parametric modulators of the first stage onset regressor with previous reward and the interaction of reward and transition being our first two contrasts of primary interest. The contrast representing previous reward was used as a measurement for MF control whereas the reward × transition contrast was considered to reflect MB behaviour. On second-stage onset, we included one onset regressor for current common transition trials and for current rare transition trials and the difference of these onset regressors (common – rare) was our third contrast of interest. Analogously, we included onset regressors for current rewarded and unrewarded trials, respectively, at outcome presentation, their difference contrast (rewarded – unrewarded) being our last contrast of interest. The third and fourth contrasts of interest were used to assess whether the tryptophan intervention affected outcome or transition representation in general. For each contrast we report its main effect in the results section as a check for intervention independent task-related brain responses.

#### Region of interest analyses

Region of interests (ROIs) were selected a priori based on previous studies: ventral striatum and vmPFC, as both have been associated with MF and MB signals (Balleine and O’Doherty, 2010; Daw et al., 2006, 2011; Nebe et al., 2018; Nee et al., 2013; Wager et al., 2004), and dlPFC due to its known association with MB behaviour (Smittenaar et al., 2013). The ROIs were chosen similar to Kroemer et al (2014) and are visualised in the Supplemental Figure S3. We extracted the mean BOLD signal in the ROIs for each session and each of our four contrasts of interest. We compared the values of the extracted ROI activation in a repeated-measures ANOVA with tryptophan intervention as within-subject predictor. In this analysis, we considered all values *p* < 0.05 (two-tailed) as statistically significant and report the within-subject contrasts and η_p_^2^ as measurement for effect size.

#### Exploratory whole-brain analyses

Further exploratory second-level statistics were conducted with the tryptophan interventions (ATL, BAL, ATD) as a repeated measures factor for the same parametric contrast images than in the first-level analysis. We set up whole-brain analyses as *F*-tests, with the intervention as repeated measures factors. These additional analyses were performed at a voxel-based threshold of *p*_uncorr._ < 0.001 and a cluster-based, family-wise error (FWE) corrected threshold of *p*_FWE_ < 0.05.

## Results

### Main effects of the two-stage task

#### Behavioural data

**Figure 2.**
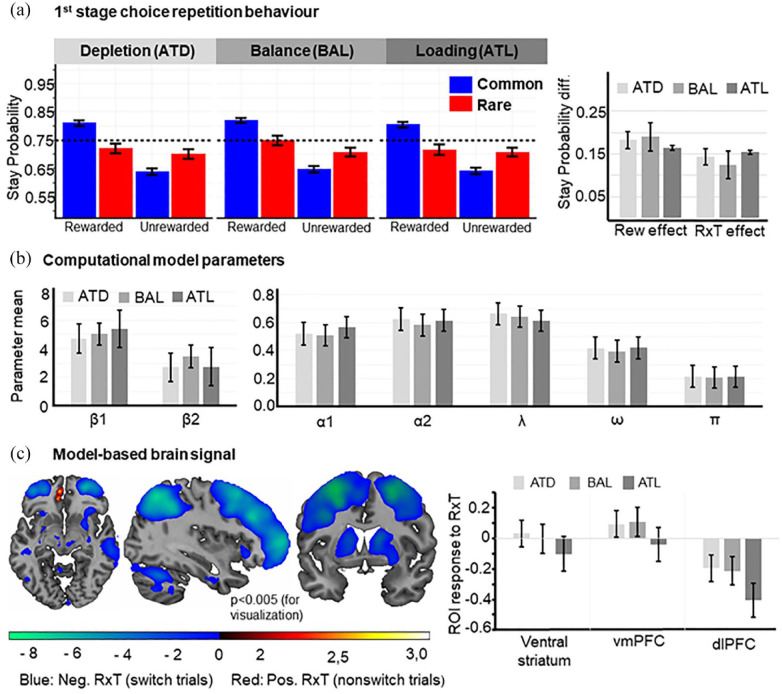
Behavioural and brain effects of tryptophan interventions. (a) Impact on stay-switch probabilities: Tryptophan levels had no substantial influence on the pattern of first stage choice repetition rates (left). Neither model-free nor the model-based (MB) scores were influenced by tryptophan (right). Error bars: Standard error of the mean (SEM). (b) Effect of intervention on model parameters derived from computational model: We did not observe an effect of the tryptophan intervention on any model parameter. Error bars: SEM. (c) MB brain signal. Coordinates: *X* = 36, *Y* = 5, *Z* = −8. In stay trials (red scale), we found activation in the left vmPFC, which expand to the ventral striatum. In switch trials (blue scale), we saw increased activation in dlPFC, striatum, insula, pallidum, motor and premotor areas. Bar plots visualize the brain response in the regions of interest for the three conditions. The only significant difference between the ATD/ATL and BAL condition was observed in the vmPFC, were the BOLD signal under ATL was decreased compared to the BAL condition (*p* = 0.021). Error bars: SEM. Uncorrected *p* < 0.005 for visualization purpose only. ATD: acute tryptophan depletion; ATL: acute tryptophan loading; BAL: baseline; BOLD: Blood-Oxygenation-Level Dependent; dlPFC: dorsolateral prefrontal cortex; vmPFC: ventromedial prefrontal cortex.

Initially, we explored the main effects of the task by analysing the frequency of repeating the first stage choice of the previous trial. This analysis does not premise any computational assumptions and is consequently unbiased. Regarding the task logic, a substantial main effect of reward indicates a MF choice strategy, whereas a MB strategy is reflected by a significant reward × transition interaction. Across all conditions we observed that first stage choice repetitions were significantly more frequent after obtaining a reward in the previous trial (i.e. main effect of reward, *F*(1,97) = 82.250, *p* < 0.001, η_p_^2^ = 0.459), which indicates a MF strategy. The type of transition between first and second stage did not affect repetition of the next first stage choice, *F*(1,97) = 1.898, *p* = 0.171, η_p_^2^ = 0.019. On the other hand, first stage repetition rate was higher when the previous choice was rewarded after a common transition, while we observed more switching after being rewarded following a rare transition in the previous trial (i.e. reward × transition interaction, *F*(1,97) = 69.512, *p* < 0.001, η_p_^2^ = 0.417). Taking the previous transition into account is indicative of a MB control strategy. Summing up, the behaviour of our subjects was based on a mixture of MB and MF control, which is perfectly in line with previous reports (Daw et al., 2011). To further quantify the balance between MB and MF control we also fitted a computational model to the data. To this end, we used the well-established model by Daw and colleagues (2011), in which the balance between MF and MB behaviour is conceptualized by the parameter omega (ω), which is zero for pure MF behaviour and one for pure MB control. We observed a mean ω of 0.41 (SD = 0.29), which lies in the range reported by previous literature. All model parameters are visualized in Figure 2(b).

#### FMRI data

To check whether the two-stage task elicited expected brain responses we analysed the main effects of the task with respect to the (1) MF signal, (2) MB signal, (3) transition signal, and (4) the outcome signal. Receiving compared to not receiving a reward in the previous trial (MF signal) was reflected by increased brain activity at the onset of first stage of the following trial in fronto-striatal areas, especially in the ventral striatum and the vmPFC. On the other hand, reward omission (compared to receipt) in the previous trial was associated with increased brain responses in the dlPFC, insula, thalamus, and dACC at onset of the following trial.

For the MB signal, we found increased BOLD activity in the left ventral striatum and a small cluster extending from the left ventral striatum to the vmPFC after rewarded common and unrewarded rare trials (i.e. positive reward-transition interaction), whereas unrewarded common and rewarded rare trials (i.e. negative reward-transition interaction) led to an increased activity in dlPFC, insula, pallidum, motor and premotor areas (Figure 2(c)).

For the transition signal at the onset of the second stage (i.e. common – rare) of the task we observed BOLD activation in value-tracking areas such as the vmPFC, the ventral striatum and the dACC after a common transition. Contrariwise, after rare transitions we found higher activation in the intraparietal sulcus and the lateral prefrontal cortex.

For the outcome signal (i.e. rewarded – unrewarded), we observed BOLD activity in reward processing areas such as fronto-striatal networks (peak activation ventral striatum bilateral).

### Intervention effects on MF and MB control

#### Behavioural data

Next, we assessed the effect of the tryptophan interventions on the observed stay-switch behaviour. Neither did our tryptophan intervention influence MF (i.e. intervention × reward interaction, *F*(2,194) = 0.645, *p* = 0.526, η_p_^2^ = 0.007, *BF*_10_ = 0.064) nor MB behaviour (i.e. intervention × reward × transition interaction, *F*(2,194) = 0.834, *p* = 0.436, η_p_^2^ = .009, *BF*_10_ = 0.080), nor the effect of transition alone (intervention × transition interaction, *F*(2,194) = 0.410, *p* = 0.664, η_p_^2^ = 0.004, *BF*_10_ = 0.054). Together, this indicates that tryptophan levels have no significant effect on first stage choice repetition (Figure 2(a)). Moreover, the Bayes factors (BF)indicate strong evidence for the null hypothesis. Echoing the results from the factorial analyses of first stage choice repetition, we observed that the weighting parameter ω, was not affected by the trypthophan intervention (*F*(2,194) = 0.298, *p* = .742, η_p_^2^ = 0.003), and *BF*_10_ = 0.048 indicates strong evidence for H0. As visualised in Figure 2(b) all other included model parameters indicating stay-switch behaviour also remained unaffected by troptophan levels (all *p* > 0.065, all *BF*_10_ < 0.271).

#### FMRI data

First, we tested in pre-defined ROIs (vmPFC, ventral striatum, dlPFC) whether the tryptophan intervention affected brain responses. To further examine possible intervention effects in other brain areas, we conducted exploratory whole-brain analyses.

Regarding the MF signal, different tryptophan levels yielded no change in BOLD activity within the three pre-defined ROIs, (all *p* > 0.71, all *BF*_10_ < 0.054). The exploratory whole-brain analysis with an *F*-test across all tryptophan levels also did not show any significant changes in brain activity (no clusters with threshold cluster-based *p*_FWE_ < 0.001).

Regarding the MB signal (compare Figure 2(c)), the tryptophan interventions significantly modulated the BOLD signals in ventral striatum (*F*(2,174) = 4.046, *p* = 0.019, *η*_p_^2^ = 0.044, *BF*_10_ = 0.545) and vmPFC (*F*(2,174) = 3.712, *p* = 0.026, *η*_p_^2^ = 0.041, *BF*_10_ = 0.755), albeit only on an anecdotal evidence level in favour of H1. Pairwise comparisons between the estimated marginal means of each intervention type in the ventral striatum revealed neither a difference between ATD and BAL (*p* = 0.45, *BF*_10_ = 0.219) nor ATL and BAL (*p* = 0.05, *BF*_10_ = 0.906). In comparison, in the vmPFC we observed a significant decrease of the BOLD signal in the ATL compared to the BAL condition (*p* = 0.021, *BF*_10_ = 0.335). There was no difference on the BOLD effect in the vmPFC between ATD and BAL (*p* = 0.829, *BF*_10_ = 0.167). In the dlPFC, there was no significant change in BOLD activity between the interventions (*F*(2,174) = 2.358, *p* = 0.098, *η*_p_^2^ = 0.026, *BF*_10_ = 0.473) with the *BF* indicating anecdotal evidence for H0. To explore whether an effect of the interventions can also be found outside our ROIs, we computed a whole-brain *F*-test. However, this analysis revealed no significant change in neural activity (cluster-based *p*_FWE_ < 0.05).

In the three ROIs, the representation of transition at the onset of the second stage of the task or the outcome signal during the presentation of reward remained unaltered by tryptophan levels (all *p* > 0.12) and no changes on the BOLD effect could be found in the exploratory whole-brain analyses.

## Discussion

We investigated whether changes in brain serotonin levels (induced by dietary tryptophan manipulations within subjects) affect goal-directed and habitual control (assessed with a two-stage sequential Markov decision task) in a large group of 98 healthy participants. In contrast to our hypotheses and previous reports, we did not observe any behavioural effect of the tryptophan intervention on MB or MF control. This null finding was mirrored by the fMRI analyses that revealed no significant impact of the interventions in any of three hypothesized ROIs (ventral striatum, vmPFC, dlPFC) on the representation of MF control. Despite the lack of any behavioural effect, the MB fMRI signal in the vmPFC was lower during tryptophan loading compared to the control condition.

The factorial analysis of stay probabilities of choices at the first stage indicates that neither MF nor MB control were significantly modulated by the dietary depletion or elevation of tryptophan levels. In line with this, the analysis based on fitting a computational model to the behavioural data also showed that the balance between both control modes was not affected by the levels of tryptophan. In fact, Bayes statistics revealed strong evidence against an effect of the intervention (BFs ranged from 13 to 21 for the null hypothesis). Additional analyses revealed that neither genetic variation of *5-HTTLPR* nor intervention order might have obscured an effect of our dietary intervention. Obviously, these findings are at odds with two previous studies by Worbe et al. (2015, 2016). In their first study, Worbe et al., (2015) used a slip-of-action task, in which they found a behavioural shift towards habitual control after tryptophan depletion. Even though the two-stage task and the slip-of-action task are both meant to measure the balance between MB and MF control, the operationalization of the control mechanisms differs between both tasks (Friedel et al., 2014; Sjoerds et al., 2016), which might explain the discrepancy between their finding with the slip-of-action task and our findings with the two-stage task. In their second study, Worbe et al. (2016) used the two-stage task with a reward and a punishment condition. Here, they observed, that ATD reduced MB behaviour in the rewarded condition but raised MB control in the punishment condition. The study was performed with a relatively small sample size (*n* = 44) and a between-subject design that bears a higher risk to overestimate effect sizes. Moreover, they investigated a young and probably well-educated sample (mean intelligence quotient (IQ) > 120), whereas our participants were drawn from the general population and were older.

It is unlikely, that differences in the effectiveness of our dietary tryptophan intervention have caused the inconsistent finding. We measured blood tryptophan levels in all participants and demonstrated a significant reduction in tryptophan blood levels after depletion as well as an increase of tryptophan levels in the loading condition (Neukam et al., 2018). Furthermore, we adapted the amount of amino acids to participants’ body weight as previously described by Biskup et al. (2012). As described in the Supplemental Material, the obtained levels of tryptophan and LNAAs were comparable with the previous literature such as the study by Dougherty et al (2008).

Summing up, we cannot clarify the mismatch between this study and the previous findings. However, we conclude that our study provides strong evidence for no influence of acute changes of brain serotonin levels on MF and MB behaviour in the two-stage task. Certainly, our findings are limited to a rewarded environment, and we cannot provide any evidence for a punishment condition.

In line with the behavioural findings, tryptophan levels did not modulate the MF brain signal. In consideration of the Bayes statistic, we only observed a very limited effect of the intervention on the MB brain signal in the vmPFC. Noticeably, this decrease in the BOLD signal yielded no behavioural relevance. Given our hypothesis, we expected that tryptophan loading would lead to an increase, not a decrease of the MB signal in the vmPFC. Even though, one might argue that changes in task behaviour were subtle and could have become evident in the more sensitive fMRI analyses, the results are contradictory to our previous hypothesis and are difficult to interpret.

While we have observed serotonergic effects on the functional connectivity of the brain during resting-state (Deza-Araujo et al., 2019) the impact of serotonin on decision-making remains elusive as we did not find behavioural effects in this and previous studies that measured different facets of decision-making, such as intertemporal and risky choice behaviour (Neukam et al., 2018, 2019). Therefore, this study adds to the growing overall picture that acute changes of serotonin levels alone have only a minor influence on decision-making.

Taken together, our data does not support the hypothesis that transient changes in brain serotonin levels – neither decreased nor increased levels – affect MF or MB control in a sequential decision-making task. In conclusion, global changes of serotonin seem to be inadequate to predict changes in goal-directed and habitual behaviour. Our finding does not exclude that regional changes in serotonin levels or the activation of certain 5-HT receptor subtypes, as it was shown in animal studies using optogenetics (Ohmura et al., 2021), affect volitional control. Comparable procedures are not feasible in human studies currently, but might be in the future.

## Supplemental Material

sj-pdf-1-jop-10.1177_02698811231216325 – Supplemental material for Arbitration between model-free and model-based control is not affected by transient changes in tonic serotonin levels Click here for additional data file. Supplemental material, sj-pdf-1-jop-10.1177_02698811231216325 for Arbitration between model-free and model-based control is not affected by transient changes in tonic serotonin levels by Maximilian D. Gilger, Lydia Hellrung, Philipp T. Neukam, Nils B. Kroemer, Stephan Nebe, Shakoor Pooseh, Yacila I. Deza-Lougovski and Michael N. Smolka in Journal of Psychopharmacology
