# Supplementary material for: Arbitration between model-free and model-based control is not affected by transient changes in tonic serotonin levels
Source: J Psychopharmacol. 2023 Dec 27;38(2):178–87. doi: 10.1177/02698811231216325 (PMC10863371; doi:10.1177/02698811231216325)
Supplement: sj-pdf-1-jop-10.1177_02698811231216325 – Supplemental material for Arbitration between model-free and model-based control is not affected by transient changes in tonic serotonin levels [file sj-pdf-1-jop-10.1177_02698811231216325.pdf]

Supplement

Methods

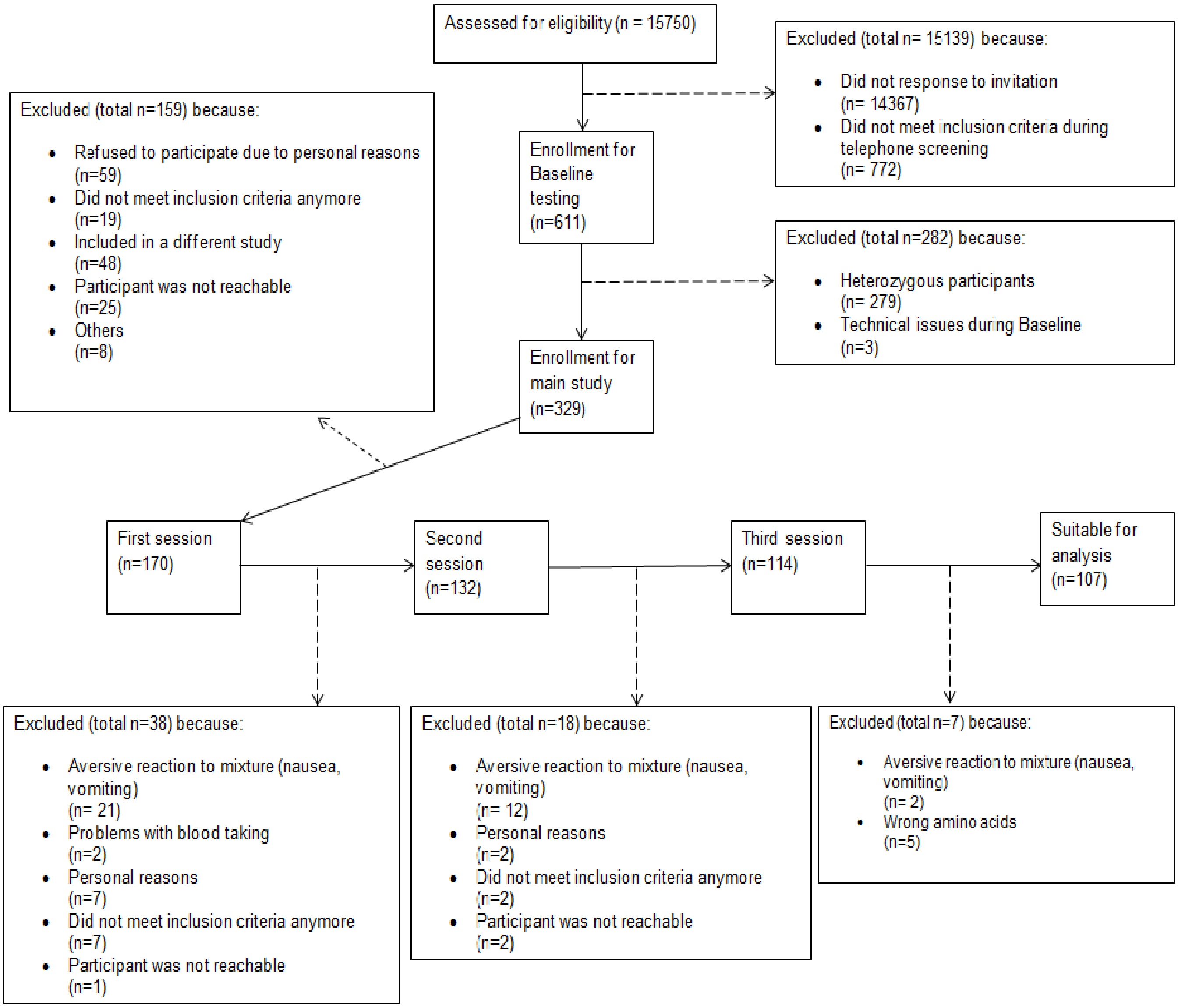

Figure S1: Recruitement workflow. A randomized sample, stratified by age (between 20 and 40 years) and gender was drawn from the registration data of the city of *[details omitted for double-anonymized peer review]* and was then invited to participate in the study. In total 1383 participants responded and were pre-screened by trained psychologists via telephone. The exclusion criteria are stated below. Due to stratification for a polymorphism in the serotonin transporter gene-linked polymorphic region (5-HTTLPR), we excluded all heterozygous participants (n=279). In total 329 participants were eligible for the study, 170 gave written consent to participate and were included in the study. Only 107 participants finished all three sessions.

Exclusion criteria for screening and during study

- Any conditions posing safety issues with the fMRI scan (e.g. metallic implants, cardiac pacemakers, pregnancy etc.)
- Corrected binocular visus below 0.8 (to ensure sufficient view of the visual cues in the paradigm)
- Any psychiatric disorder requiring pharmacologic treatment within the last year
- Life time history of one of the following: organic psychiatric disorders (F0), opiate, cocaine, stimulant, hallucinogen, inhalant, or poly-substance dependence, schizophrenia or related disorders (F2), affective disorders (F3)
- Current somatic disease requiring treatment with drugs affecting the central nervous system

We further excluded participants, who failed to show up sober, who did not tolerate the mixture (strong aversion, vomiting) or who actually planned to get pregnant. Light and heavy smokers were accepted. We asked them not to smoke at least one hour before the fMRI session. The participants were allowed to take regular medication (except psychiatric and antiepileptic, opiates or any other drugs affecting the brain).

Exclusion for behavioural analyses:

For analysis, we had to exclude five participants due to task unresponsiveness (more than 30% missing trials in at least one session), two due to technical issues during data acquisition, and two due to an intervention randomization error in one session. This resulted in a sample of 98 participants for behavioural analysis.

Exclusion for fMRI analyses because of bad data quality:

For fMRI analysis, we excluded another 10 participants due to insufficient data quality and hence analysed a sample of 88 participants. We initially performed a visual quality control of the acquired data and excluded 5 participants with strong unrepairable artifacts such as severe ghosting, spiking or cut off brain images. All other fMRI images were repaired with the ArtRepair toolbox. The repair was data driven (i.e. the extent of repair differed due to actual data quality). We excluded four participants who had more than 30% repaired slices

in at least one session and one further participant with a very high scan-to-scan motion. This results in a sample of 88 participants for the fMRI analyses.

Demographic data

The sample used for the behavioural analyses included 61 male (62,2%) and 37 female (37,8%) participants, aged 20 to 42 years (Mean: 32.2 years, SD = 6.1 years). During the baseline visit, we measured the participants body weight, which was needed to calculate the amino-acid dosage (cf. Supplement section "Tryptophan intervention and blood sampling"). The participant's weight ranged between 50 and 139 kg, with a mean body weight of 75.1 kg (SD = 15.8 kg). The body mass index was in mean 24,2 kg/m^2. As expected females were more lightweight (Mean weight = 65.0 kg, SD = 9.8 kg) as men (Mean weight = 81.2 kg, SD = 15.7 kg). As seen in table S1, 53 participants (54%) had an academic degree or higher. A majority of 76 % were non-smokers or ex-smokers (nicotine abstinence for at least three months prior to baseline).

			Highest reached education				
			vocational training	technical diploma	academic degree	Ph.D or higher	Total
Sex	male	Number	12	10	26	11	59
		Percentage %	20.3%	16.9%	44.1%	18.6%	100.0%
	female	Number	15	6	10	6	37
		Percentage %	40.5%	16.2%	27.0%	16.2%	100.0%
Total		Number	27	16	36	17	96*
		Percentage %	28.1%	16.7%	37.5%	17.7%	100.0%

Table S1: Percentage of highest reached education. For two participants the data is missing.

The sample used for the fMRI analyses included 52 male (59%) and 36 female (41%) participants, aged 20 to 42 years (Mean 31.8 years, SD = 6,1 years). 50 participants (57%) had an academic degree.

Genotyping

It was hypothesized that serotonergic functioning is influenced by the expression of a serotonin transporter in the cell membrane. This expression is genetically controlled (Meltzer and Arora, 1988). The gene coding for the serotonin transporter is the SLC6A4 gene on the long arm of chromosome 17. This gene has a short (S) and a long (L) allele. Homozygous carriers of the short allele are supposed to have a lower transcription rate (and therefore expression) of the serotonin transporter than homozygous carriers of the long allele. Recent studies found that the L allele itself is varied through an adenine-guanine substitution (SNP rs25531) resulting in a triallelic locus (S, L_A, L_G). It could be shown that the L_G version has the same transcriptional properties as the S allele in vitro (Hu et al., 2006). Hence, we can subsume the different genotypes according to their transcriptional efficiency into three categories: High transcriptional efficiency (L_A/L_A), low transcriptional efficiency (S/S, S/L_G, L_G/L_G) and the heterozygous participants with a mixed serotonin transporter expression (S/L_A, L_A/L_G). Here, we refer to this classification as functional genotype. We decided to use a triallelic locus for the genotype. There was a misassignment of ten participants to the study group. Nine participants are only homozygous in a biallelic testing but not in a triallelic locus (L_A/L_G) and should not have been included. One participant was heterozygous in both loci (S/L_A) and was only invited to the main study due to a miscommunication with the laboratory. Including these participants does not influence the interpretation of our results.

All participants were genotyped at the time of the baseline visit. The samples were collected in 9 ml tubes, laminated with ethylenediaminetetraacetic (EDTA) (Sarstedt, Nümbrecht, Germany) and then immediately frozen in a -80°C refrigerator. The distribution of the different allelic variations is shown in Figure S2.

A

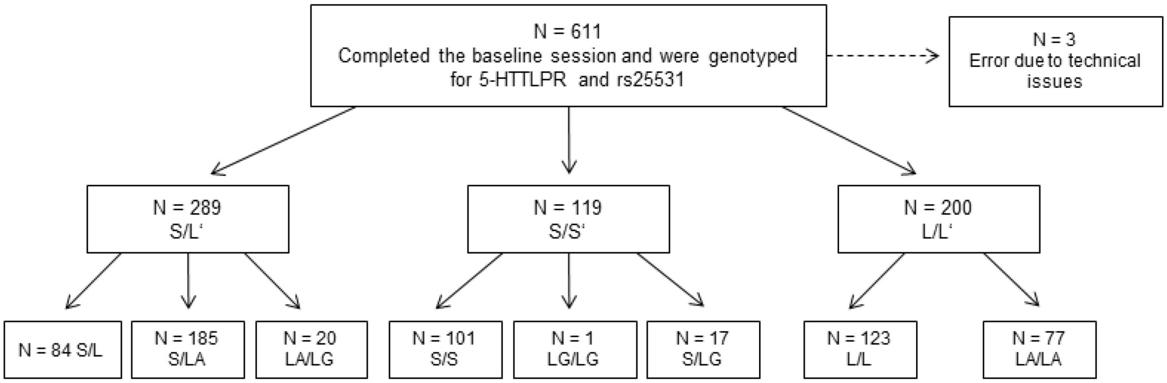

B

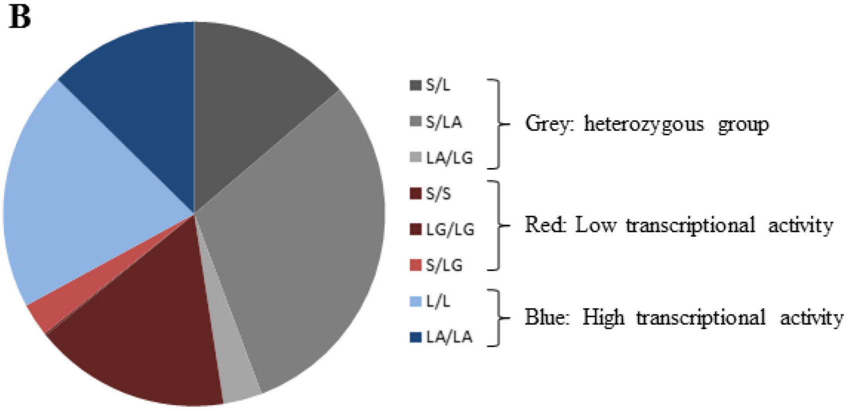

C **Triallelic genotype**

	Frequency	Percentage
S/S	38	38.8
S/LG	6	6.1
S/LA	1	1.0
LG/LG	0	0.0
LG/LA	9	9.2
LA/LA	44	44.9

D **Functional genotype**

	Frequency	Percentage
S/S	44	44.9
S/L	10	10.2
L/L	44	44.9

Figure S2: Distribution of different genetic variations in our sample. **Frequency of allelic variations in all genotyped participants (A):** S: short allele; L: long allele. **Pie chart of allelic variations in all genotyped participants (B):** Heterozygous participants were excluded from the study. Participants with S/S, L_G/L_G or S/L_G genotype were merged to a group with low transcriptional activity of the serotonin transporter. L/L and L_A/L_A participants were considered as group with high transcriptional activity of the serotonin transporter (functional genotype). **Tables with distribution of genotypes in the final sample for behavioral analyses (n=98) (C +D).**

Tryptophan intervention and blood sampling

We asked our participants to abstain from protein rich foods 24 hours before beginning of the study and having fasted overnight. All amino acid mixtures contained the same amount of (body weight adapted) large neutral amino acids (LNAA but differed in the amount of tryptophan. The amino acids were from the outset body weight adapted (as detailed in Table S2 below), which results in a total amount of 48.75 g of large neutral amino acids (LNAA) for a 75 kg participant (mean body weight of our participants). As females were more lightweight in our study they received in general a smaller amount of LNAAs (42.25 g for a 65 kg female participant). In the depletion condition the mixture was completely tryptophan-free, for balance condition 7 mg/kg body weight tryptophan (.525 g for a 75 kg participant) were added, whereas in the loading condition we added 70 mg tryptophan per kg body weight (5.25 g for a 75 kg participant). The three different amino acids were provided by a commercial manufacturer (Amino-Factory, Lindenberg, Germany) in powder form and was dissolved in water and sprite (without carbonic acid) about 1 hour before study begin. All included amino-acids are listed in Table S2 below. We instructed our participants to drink the mixture as fast as possible. The mean time needed for drinking the mixture was 11 minutes (SD: 8 minutes).

Amino Acids	g/kg mixture	g/kg body weight	
**Phenylalanine**	203.1	0.132	∑LNAA = 0.650 g/kg body weight
**Leucine**	203.1	0.132	
**Isoleucine**	129.2	0.084	
Methionine	76.9	0.050	
**Valine**	147.7	0.096	
Threonine	92.3	0.060	
Lysine	147.7	0.096	
Tryptophan added Depletion/Balance/Loading	0/10.7/97.2	0 / 0.007 / 0.07	

Table S2: Composition of the amino acid mixture. The amino acid mixture consists of the same amount of LNAAs in each condition (depletion, balanced, loading). The amount of tryptophan varies between the conditions and is added separately to the mixture. Bold: All amino acids that are tryptophan competitors.

To ensure the effectiveness of the intervention, we performed a blood sampling four times during the study. The first blood taking was performed before ingestion of the amino-acids as a reference (T0), then 1h later (T1), shortly before the fMRI-scan (approximately 3 - 3,5 h after ingestion of the amino-acids, T2) and after the fMRI scan (approximately 6 h after drinking the mixture, T3). In case the blood taking was not possible or only partially possible, we continued the study without blood takings. The samples were collected in 9 ml tubes, laminated with ethylenediaminetetraacetic (EDTA) (Sarstedt, Nümbrecht, Germany). Immediately after collection, the sample was centrifuged at 4000 g and 4°C for 10 minutes and then the plasma was separated and stored in the -81°C refrigerator until analysis.

Analyses were conducted at the Department of Chemistry and Food Chemistry of the Technische Universität Dresden as described previously (Henle et al., 1991).

For the analysis of the tryptophan blood levels, we used the same procedure as previously described in the paper by Neukam et al (2018). In short, we calculated the ratio between the tryptophan plasma levels and the other large neutral amino acids (ΣLNAA), which we consider more relevant for the interpretation of our results as the tryptophan levels alone (cf. Fernstrom and Wurtman, 1971). To account for intraindividual differences in baseline blood levels, all obtained blood levels were normalised by subtracting the reference measure (T0) from the other time points (T1, T2, T3). We integrated over all time points to compute area under the curve (AUC) scores, which were then entered into repeated-measures ANOVA as dependent variable with intervention as a within-subject factor.

Computational model:

We set up the model as detailed by (Otto et al., 2013). The paradigm consists of three states. One first stage (grey boxes, denoted as S) and two possible second stages (either pair of colored boxes, denoted as S'_1 and S'_2). For each stage, the participant can choose between two actions (denoted as a_1 and a_2). The transition contingency between the first-stage S and the second-stage **S'** is predefined as followed:

$$P(S'_1|S,a_1)=0.7,\ P(S'_2|S,a_2)=0.7 \text{ or } P(S'_1|S,a_2)=0.3,\ P(S'_2|S,a_1)=0.3$$

and remains fixed during the whole task. After each trial the participant is rewarded according to a diffusive probability which assigns non-zero probabilities within the boundaries of 0.25 and 0.75 to each second-stage action.

The model is computed as a hybrid reinforcement learning model with weighted components for model-free (MF) and model-based (MB) controls. The MF system is characterized by updating state-action values at each trial by temporal difference learning, whereas the MB component takes the transition contingencies between the two stages into account.

Noticeably, on the second stage there is only a MF component which is driven by the expected reward in the current trial (denoted as r_t) and the current state-action-value $Q(S'_t,a)$. Thereby follows for the second-stage:

$$Q\,(S'_t,a)=Q\,(S'_{t-1},\ a) + \alpha_2[r_t-Q(S'_{1-t},a)]$$

Here, $r_t-Q(S'_{1-t},a)$ represents the reward prediction error (RPE) and $0<\boldsymbol{\alpha_2}<1$ the learning rate at the second stage. For the first stage value updating works similar but with a different learning rate $0<\boldsymbol{\alpha_1}<1$ and an additional eligibility parameter for stage-skipping value updating $0<\boldsymbol{\lambda}<1$.

$$Q_{MF}(S_t,a)=Q_{MF}(S_{t-1},a)+\ \alpha_1[Q_{MF}(S'_{t-1},a)-Q_{MF}(S_{t-1},a)]+\ \alpha_1\ \lambda[r_{t-1}-Q_{MF}(S'_{t-1},a)]$$

The MB component learns by mapping state-action pairs to transition probabilities such as

$$Q_{MB}(S_t,a)=P(S'_1|S,a)\max Q_{MF}(S'_{1,t-1},a)+P(S'_2|S,a)\max Q_{MF}(S'_{2,t-1},a)$$

The values are connected to choices via a softmax choice rule, which maps probabilities to each action according to a combination of MF and MB components. The two components are weighted by a parameter β_{MB} and β_{MF}. The tendency to repeat the previous first-stage option is captured by the perseveration parameter π. This results in the probability for a single choice at the first stage as

$$P(a \mid S_t) = \frac{\exp[\beta_{MB} Q_{MB}(S_t, a) + \beta_{MF} Q_{MF}(S_t, a) + \pi \times rep(a)]}{\sum_a \exp[\beta_{MB} Q_{MB}(S_t, a') + \beta_{MF} Q_{MF}(S_t, a') + \pi \times rep(a')]}$$

Whereby, rep(a) is a function which equals one if the previous first-stage choice is repeated and zero if not.

fMRI data

ROI masks

The masks were selected as described by Kroemer et al (2014). In short, the masks of vmPFC, ventral striatum and dlPFC were taken from meta-analysis software (http://old.neurosynth.org/terms/ and the BrainMap database (Nielsen and Hansen, 2002)), respectively. The vmPFC mask from neurosynth.org was smoothed and parts of the ACC were removed. The mask of the dlPFC was also smoothed. The mask of ventral striatum from BrainMap has been thresholded with p>.6.

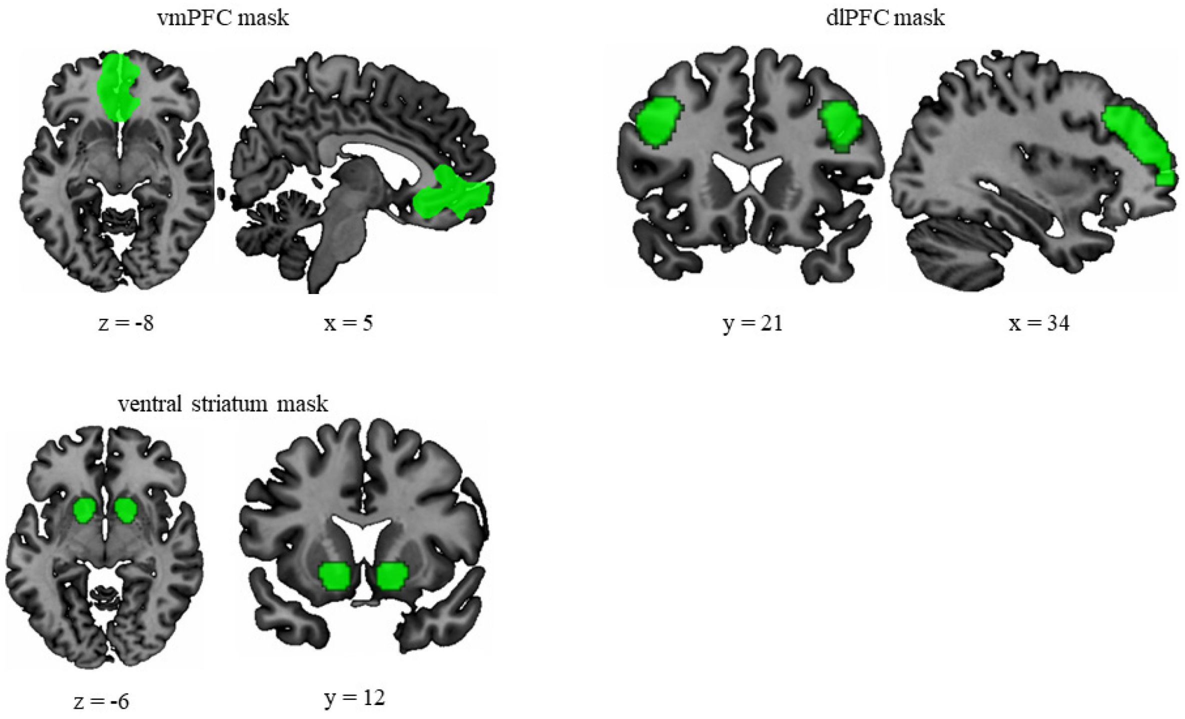

Figure S3: ROI masks

Image despiking and motion correction Procedure: For image repair we used the ArtRepair toolbox for SPM. All volumes were subjected to a bad slice correction step, which repairs slices with an unusual amount of data scattered outside the head (Threshold: T=7%) by interpolation, followed by slice time correction (reference: middle slice) and by realignment to the first volume of the run to correct for motion. Distortion correction based

on the field map was then applied to the realigned EPI images. All distortion-corrected images were consigned to a despiking (Threshold: T=4 percentage signal change to the mean image) and additional big scan-to-scan motion repair (Threshold: T=0.5mm/TR).

Results

Pharmacological results of the tryptophan intervention

As seen in Table S3 and Figure S4 the tryptophan blood levels reached its maximum 3 hours after ingestion of the amino acid mixture in the loading condition and its minimum 3 hours after ingestion in the depletion condition. In the loading condition, we observed an increase of tryptophan peak levels of 339% relatively to the baseline (pre-drink) measure. After ATD the tryptophan levels decreased by 31% relatively to the baseline measure. To check the comparability of our study and interventional procedure with other dietary tryptophan loading and depletion studies, we compared the measured tryptophan and LNAA values with the literature. Dougherty et al (2008) tested the effectiveness of either a 50 g or 100 g amino acid drink, which was based on the formulation of Young et al (1985) in 112 healthy adults. The primary findings of this study were, that either the 100 g and the 50 g formulation, led to robust changes of plasma tryptophan. As in our study, a 75 kg participant (mean weight of our participants) received 48.75 g of large neutral amino acids plus a maximum of 5.25 g tryptophan in the loading condition, we can only compare to the 50 g formulation in the Dougherty-study. In the ATD condition, Dougherty et al measured a tryptophan level of 22 μM in the 50 g total formulation, while we observed a mean tryptophan level of 22.56 μM. The sum of the tryptophan competitors was 1532 μM in the above cited paper and 1360 μM (excl. threonine, lysin and methionine) in our study. For BAL Dougherty et al measured a tryptophan level of 143 μM, while we observed a mean tryptophan level of 39.75 μM. The sum of the tryptophan competitors was 1431 μM in the above cited paper and 1334 μM (excl. threonine, lysin and methionine) in our study. For ATL Dougherty et al measured a tryptophan level of 491 μM in the 50 g total formulation, while we observed a mean tryptophan level of 147.62 μM. The sum of the tryptophan competitors was 1223 μM in the above cited paper and 1157 μM (excl. threonine, lysin and methionine) in our study. The fasting tryptophan concentration was between 56 μmol/l and 74 μmol/l in the Dougherty paper, which is slightly higher than in our study (between 32.62 and 33.62 μM/L).

The ANOVA revealed a significant main effect of the tryptophan intervention on TRP/ΣLNAA AUC values, $F_{(2,166)} = 658.99$, $p<.001$. An additional contrast analysis showed a significant decrease of TRP/ΣLNAA AUC values in the tryptophan depletion condition in comparison to balance, $F_{(1,83)} = 75.22$, $p<.001$, while we observed an increase in acute tryptophan loading compared to balance, $F_{(1,83)} = 601.63$, $p<.001$. In conclusion, our findings with the Moja-De formulation are in the same range as in the above cited paper using the Young formulation and the ANOVAs demonstrate significant changes of the TRP/LNAA ratio as a parameter for brain serotonergic availability.

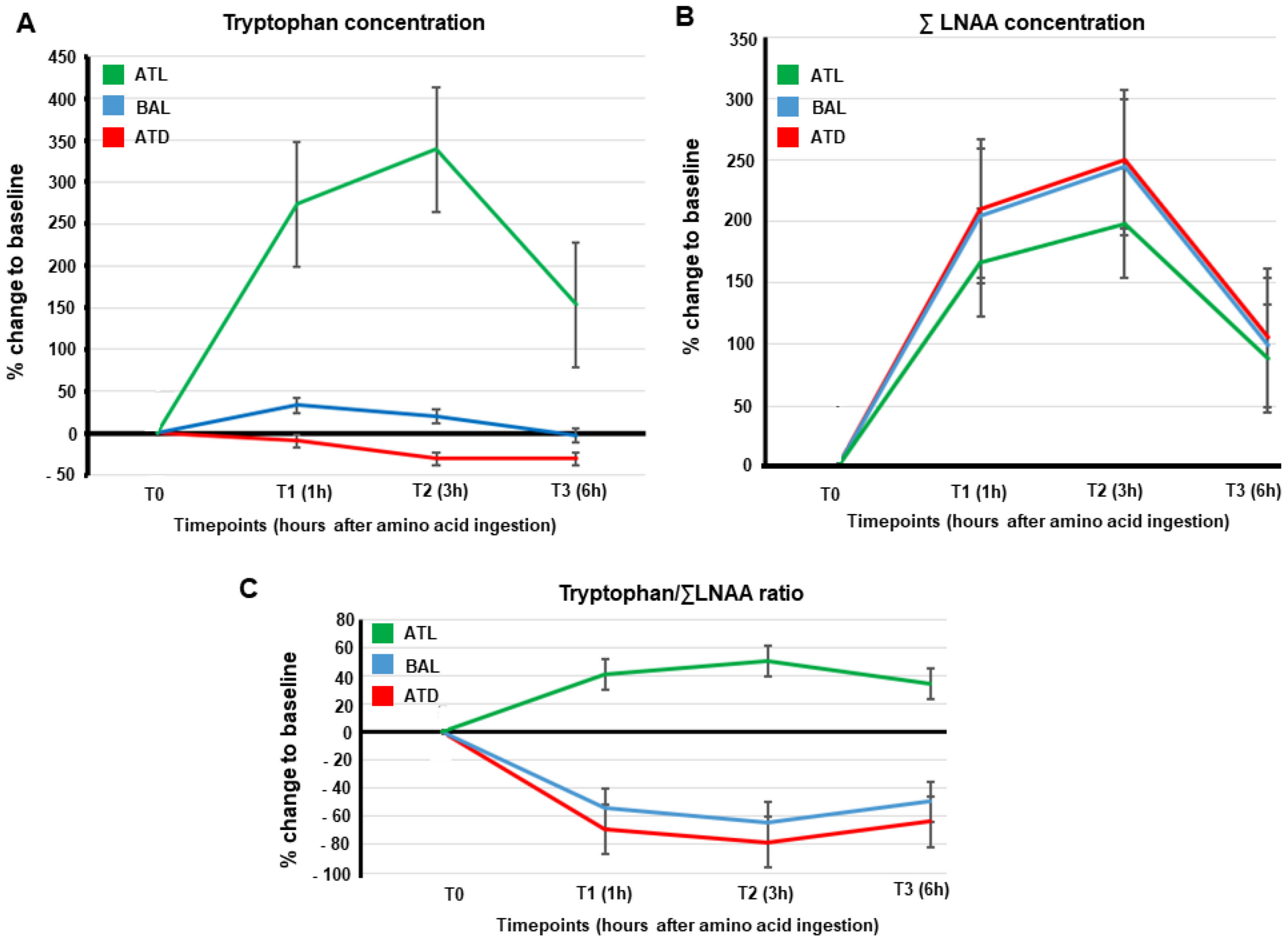

Figure S4: Pharmacokinetics of the tryptophan intervention. **(A) Percent change of blood tryptophan concentration over the time of the study in comparison to the baseline testing (before ingestion of the amino acids).** The graph shows an increase in blood tryptophan levels in the loading condition, while in the balanced and tryptophan depletion condition the tryptophan concentration remained nearly stable during the time of the study. **(B) Percent change of the concentration of the tryptophan competitors (LNAA).** Peak levels of LNAA were reached 3h after ingestion. **(C) Percent change of TRP/ΣLNAA Ratio as a measure of central serotonergic availability.** Serotonergic availability peaked three hours after ingestion of the amino acids in the acute tryptophan loading condition (ATL) or reached their minimum after three hours in the depletion condition (ATD). Error bars: Standard error of the mean.

Acute tryptophan depletion				
Timepoints (hours after amino acids ingestion)				
	T0	T1 (1h)	T2 (3h)	T3 (6h)
**Phenylalanine**	68,72<br>± 14,13	269,08<br>± 68,99	302,84<br>± 103,37	151,23<br>± 68,26
**Leucine**	105,63<br>± 29,11	301,68<br>± 71,30	331,12<br>± 114,09	189,93<br>± 65,98
**Isoleucine**	70,09<br>± 15,92	153,08<br>± 36,23	210,84<br>± 85,02	119,27<br>± 53,54
Methionine	52,94<br>± 14,98	153,64<br>± 31,55	177,70<br>± 44,88	114,80<br>± 37,35
**Valine**	143,20<br>± 26,69	480,05<br>± 102,61	515,39<br>± 134,81	335,90<br>± 85,85
Threonine	99,46<br>± 16,07	167,29<br>± 36,06	168,02<br>± 37,90	142,15<br>± 26,62
Lysine	98,08<br>± 19,78	222,66<br>± 42,76	172,50<br>± 46,89	114,01<br>± 25,22
Tryptophan	32,62<br>± 7,19	29,65<br>± 8,07	22,56<br>± 9,16	22,70<br>± 8,68
**Balanced condition**				
Timepoints (hours after amino acids ingestion)				
	T0	T1 (1h)	T2 (3h)	T3 (6h)
**Phenylalanine**	69,96<br>± 17,07	263,40<br>± 78,91	297,51<br>± 94,94	144,29<br>± 54,10
**Leucine**	103,79<br>± 19,13	296,55<br>± 77,81	324,36<br>± 86,49	183,96<br>± 58,19
**Isoleucine**	70,56<br>± 15,28	151,49<br>± 41,13	204,66<br>± 66,15	115,54<br>± 44,91
Methionine	53,77<br>± 15,71	152,01<br>± 35,56	178,58<br>± 37,36	115,61<br>± 35,72
**Valine**	143,01<br>± 25,20	469,44<br>± 117,22	508,08<br>± 112,23	328,74<br>± 82,73
Threonine	100,58<br>± 18,30	168,28<br>± 48,41	170,08<br>± 40,00	143,80<br>± 31,47
Lysine	98,98<br>± 19,24	219,07<br>± 54,96	169,93<br>± 38,94	112,49<br>± 21,25
Tryptophan	33,26<br>7,72	44,22<br>12,75	39,75<br>10,77	32,31<br>9,39
**Acute tryptophan loading**				
Timepoints (hours after amino acids ingestion)				
	T0	T1 (1h)	T2 (3h)	T3 (6h)
**Phenylalanine**	68,86<br>± 15,32	222,85<br>± 60,48	243,09<br>± 81,78	132,36<br>± 49,68
**Leucine**	105,09<br>± 19,76	261,87<br>± 66,64	282,52<br>± 88,03	175,47<br>± 60,14
**Isoleucine**	70,16<br>± 15,24	134,97<br>± 32,43	182,94<br>± 66,87	116,53<br>± 56,10
Methionine	55,45<br>± 17,45	132,57<br>± 28,57	149,48<br>± 36,94	100,78<br>± 32,48
**Valine**	143,56<br>± 29,21	414,96<br>± 99,63	448,64<br>± 118,39	306,58<br>± 81,18
Threonine	100,40<br>± 19,81	151,37<br>± 34,54	143,97<br>± 33,12	123,37<br>± 27,25
Lysine	99,92<br>± 18,85	189,74<br>± 41,02	142,94<br>± 33,99	101,58<br>± 21,11
Tryptophan	33,62<br>± 7,78	125,69<br>± 39,71	147,62<br>± 49,36	85,35<br>± 41,27

Table S3: Concentrations (µmol/l) of the amino acids for all three interventions with standard deviation. Bold: All amino acids that are tryptophan competitors.

Behavioural data: Effect of genotype and order on 1st stage choice repetition

To analyse the effect of the tryptophan intervention, we set up a repeated-measures ANOVAs with the model-free effect, and another with the model-based effect as dependent variables and intervention as the factor of interest. Furthermore, we added the 5-HTTLPR genotype and the order of the three interventions as between-subject factor to our ANOVA. This revealed no significant main effects of neither genotype, $F(1,82)= .61$, $p=.805$, nor the order of the intervention, $F(2,82)= .546$, $p=.582$ on model-free behaviour. There was also no significant interaction between genotype and intervention, $F(2,164)= 1.406$, $p=.248$.

Model-based behaviour was likewise not affected by genotype, $F(1,82)=.691$, $p=.408$ or order, $F(2,82)=1.017$, $p=.366$. The interaction between genotype and intervention also failed to reach significance, $F(2,164)=1.090$, $p=.339$.

References

Dougherty DM, Marsh-Richard DM, Mathias CW, et al. (2008) Comparison of 50- and 100-g l-tryptophan depletion and loading formulations for altering 5-HT synthesis: pharmacokinetics, side effects, and mood states. *Psychopharmacology* 198(3): 431–445.

Fernstrom JD and Wurtman RJ (1971) Brain serotonin content: increase following ingestion of carbohydrate diet. *Science (New York, N.Y.)* 174(4013): 1023–5.

Henle T, Walter H, Krause I, et al. (1991) Efficient Determination of Individual Maillard Compounds in Heat-Treated Milk Products by Amino Acid Analysis. *International Dairy Journal* 1: 125–135.

Hu XZ, Lipsky RH, Zhu G, et al. (2006) Serotonin transporter promoter gain-of-function genotypes are linked to obsessive-compulsive disorder. *Am J Hum Genet* 78.

Kroemer NB, Guevara A, Ciocanea Teodorescu I, et al. (2014) Balancing reward and work: Anticipatory brain activation in NAcc and VTA predict effort differentially. *NeuroImage* 102(P2). Academic Press: 510–519.

Meltzer HY and Arora RC (1988) Genetic control of serotonin uptake in blood platelets: A twin study. *Psychiatry Research* 24(3): 263–269.

Neukam PT, Kroemer NB, Deza Araujo YI, et al. (2018) Risk-seeking for losses is associated with 5-HTTLPR, but not with transient changes in 5-HT levels. *Psychopharmacology* 235(7): 2151–2165.

Nielsen F and Hansen L (2002) Automatic anatomical labeling of Talairach coordinates and generation of volumes of interest via the BrainMap database. *Neuroimage* 16.

Otto AR, Raio CM, Chiang A, et al. (2013) Working-memory capacity protects model-based learning from stress. *Proceedings of the National Academy of Sciences of the United States of America* 110(52): 20941–20946.

Young SN, Smith SE, Pihl RO, et al. (1985) Tryptophan depletion causes a rapid lowering of mood in normal males. *Psychopharmacology* 87(2). Springer-Verlag: 173–177.
